# Autolyzed yeast and sodium butyrate supplemented alone to diets promoted improvements in performance, intestinal health and nutrient transporter in weaned piglets

**DOI:** 10.1038/s41598-024-62551-9

**Published:** 2024-05-24

**Authors:** Amanda Medeiros Correia, Jansller Luiz Genova, Sung Woo Kim, Fernanda Fialho Abranches, Gabriel Cipriano Rocha

**Affiliations:** 1 Muscle Biology and Nutrigenomics Laboratory, Department of Animal Science, Universidade Federal de Viçosa, Minas Gerais, Brazil; 2 Department of Animal Science, North Carolina State University, Raleigh, NC 27695 USA

**Keywords:** Autolyzed yeast, Gut health, Performance, Piglets, Nucleotides, Sodium butyrate, Animal physiology, Immunology, Molecular biology, Biomarkers, DNA

## Abstract

This study investigated the effects of supplemental nucleotides, autolyzed yeast (*Saccharomyces cerevisiae*), and sodium butyrate in diets for nursery pigs on growth performance, diarrhea incidence, blood profile, intestinal morphology, mRNA expression of nutrient transporters, inflammatory markers, antioxidant profile, and tight junction proteins in the small intestine. One hundred eighty 21-day-old pigs (5.17 ± 0.57 kg) were assigned in a randomized block design to 1 of 4 dietary treatments: (1) CON: control, basal diet, (2) NUC: CON + nucleotides, (3) YSC: CON + lysed yeast *S. cerevisiae*, (4) ASB: CON + acidifier sodium butyrate. Pigs were fed for 24 days, phase 1 (21–32 days) and 2 (32–45 days). During phase 1, YSC and ASB improved average daily gain (ADG) and feed conversion (FC) compared with CON. At the overall period, ASB improved ADG and YSC improved FC compared with CON. The NUC diet did not affect growth performance. The ASB increased ileal villus height compared to CON. The YSC and ASB reduced the number of Peyer’s patches in the ileum compared with CON. The YSC increased mRNA expression of nutrient transporters (SMCT2, MCT1, and PepT1), tight junction proteins (OCL and ZO-1), antioxidants (GPX), and IL1-β in the jejunum compared with CON. The ASB increased mRNA expression of nutrient transporters (SGLT1 and MCT1), tight junction proteins (OCL and ZO-1), and antioxidants (GPX and SOD) compared with CON. In conclusion, autolyzed yeast and sodium butyrate promoted growth performance by improving the integrity of the intestinal barrier, the mRNA expression of nutrient transporters, and antioxidant enzymes in the jejunum of nursery pigs whereas supplementation of nucleotides did not show such effects.

## Introduction

Weaning is considered the “critical window” in the piglet’s life because it is associated with several stress factors, such as loss of contact with the mother and original litter, solid diet, environmental and structural changes, and the establishment of a new hierarchy^1,2^. Abrupt separation from the sow and dietary transition are critical events causing intestinal challenges and compromising immune functions affecting growth of pigs^3,4^. Dietary interventions with various functional additives have been introduced and implemented to cope with challenges to the intestine and immune functions upon weaning. Nucleotides^5,6^, yeast^7,8^ and organic acids^9,10^ have appeared on the market as feeding strategies to improve intestinal health and, consequently, promote better growth performance of weaned piglets. Globally, their use as in-feed additives in pig diets has become more frequent, especially during the weaning period^11,12^. However, factors such as the form of supplementation, the type of additive, and the site of action make each application unique and complex in animal responses and should be better understood in studies involving pigs fed different feed additives^13–15^.

Nucleotides are monomers that serve as building blocks of nucleic acids (DNA and RNA) and are synthesized by animals through the de novo pathway or the salvage pathway. These monomers function as physiological mediators, coenzyme components, and contributors to cell growth and division and are crucial to the rapid proliferation of intestinal mucosa cells and immune cells^16,17^. Thus, the supplementation of nucleotides to the diet can bring benefits in periods of rapid growth and development (e.g. digestive organs, immune systems, cell renewal) helping in circumstances associated with intestinal injuries and stress, such as post-weaning period^5,6^.

The lysed yeast (*S. cerevisiae*) contains in the cell wall a complex polymer and is composed of β-glucans, α-mannans, mannoproteins, and a minor component of chitin^18^, in addition, the cytoplasm contains B complex vitamins, highly digestible proteins and free amino acids^15^. However, monogastric animals do not have enzymes to digest polysaccharides such as those present in the yeast cell wall, therefore, the breakdown of yeast cell wall by lysing would improve the bioavailability of yeast components for animal nutrition^7^. The major polysaccharide constituents of the yeast cell wall, β-glucans and α-mannans, have been recognized to be capable of modulation of the mucosa immune system, promoting beneficial effects on pig health (e.g. inhibition of pathogen adhesion to gastrointestinal epithelial tissue and stimulation of immunocompetent cells in Peyer's patches)^19,20^. Thus, yeast products, mainly based on *S. cerevisiae*, are widely used as feed additives.

Organic acids (e.g. sodium butyrate) act by reducing the pH of digesta in the gastrointestinal tract, stimulating the activity of digestive enzymes, inhibiting the proliferation of pathogenic bacteria, and improving the digestibility of nutrients^9,21^. Sodium butyrate is a short-chain fatty acid that acts by improving villus height, decreasing crypt, and improving the expression of tight junction protein in the gut^14^. As a result, it beneficially affects the reabsorption of fluids and electrolytes which is associated with reduced diarrhea^22^, moreover modulating intestinal permeability will reduce translocation of intraluminal toxins and antigens from the lumen into subepithelial tissues and systemic blood circulation^23^. Together with improving gut health, the ability of sodium butyrate to alleviate oxidative stress and improve immunological profile will result in improved pig weight gain^14,22,24^. However, free organic acids dissociate and lose most of their antibacterial capacity before reaching the distal part of the digestive system^25^. Therefore, sodium butyrate in encapsulated form allows progressive reach to the distal parts of the gastrointestinal tract without being fully dissociated, because the product is gradually released compared to an uncoated product^10^.

In view of the above, the hypothesis of the study was that the dietary supplementation of purified nucleotide, lysed yeast (*S. cerevisiae*), or encapsulated sodium butyrate would improve the growth performance of weaned piglets by beneficially stimulating the immune response and supporting intestinal physiological and health functions. Therefore, the objective of this study was to evaluate the effects of purified nucleotide, lysed yeast, and sodium butyrate in diets for nursery pigs on growth performance, diarrhea incidence, blood profile, intestinal morphology, mRNA expression of nutrients transporter, inflammatory markers, antioxidant profile, and tight junction proteins.

## Results

### Growth performance

During phase 1 (21–32 days), pigs fed YSC or ASB diets showed (*P* < 0.05) improvements in ADG, FC, and BW compared with pigs fed CON diet; however, ADFI was not affected by dietary treatments (Table 1). In phase 2 (32–45 days), pigs fed ASB diet had higher (*P* < 0.05) BW compared to pigs fed CON diet. In addition, there was a trend to increase the BW (*P* = 0.095) in pigs fed YSC diet compared to those fed CON diet. Pigs fed NUC diet tended to present lower ADG (*P* = 0.080). However, FC was not affected by dietary treatments. During the overall period, ADG was higher (*P* < 0.05) in pigs fed ASB diet than those with CON diet. In addition, FC was better (*P* < 0.05), and a trend was observed to improve ADG (*P* = 0.095) in pigs fed YSC diet compared with pigs on CON diet. Moreover, ADFI was not affected by dietary treatments.

**Table 1:** Growth performance of piglets fed diets supplemented or not with feed additives (n = 9 pens replicates per dietary treatment and 5 piglets per pen as an experimental unit).

Item^a^	Dietary treatment^b^				SEM^c^	*P*-value		
	CON	NUC	YSC	ASB		CON × NUC	CON × YSC	CON × ASB
Body weight, kg								
Initial	5.22	5.22	5.22	5.22	–	–	–	–
32 days of age	6.83	6.89	7.18	7.18	0.11	0.693	0.034	0.031
45 days of age	12.64	12.36	13.12	13.32	0.19	0.321	0.095	0.019
Phase 1, 21–32 days								
ADFI, g/day	296	286	281	302	7.02	0.319	0.133	0.546
ADG, g/day	146	152	178	178	10.08	0.687	0.035	0.030
FC, g/g	2.05	1.97	1.61	1.74	0.09	0.549	< 0.010	0.034
Phase 2, 32–45 days								
ADFI, g/day	627	600	638	668	17.35	0.263	0.678	0.105
ADG, g/day	486	454	494	510	12.57	0.080	0.674	0.187
FC, g/g	1.29	1.32	1.29	1.31	0.02	0.336	0.940	0.602
Overall period								
ADFI, g/day	449	431	448	473	10.88	0.237	0.902	0.141
ADG, g/day	309	297	329	337	8.20	0.326	0.095	0.019
FC, g/g	1.46	1.45	1.36	1.40	0.02	0.848	< 0.010	0.103

^a^Average daily feed intake (ADFI, g/day), average daily weight gain (ADG, g/day), feed conversion ratio (FC, g:g).

^b^Dietary treatment: phase 1 (21–32 days) and phase 2 (32–45 days), respectively: (1) CON: control, basal diet, (2) NUC: CON + 1 g/kg and 0.5 g/kg of nucleotides, (3) YSC: CON + 20 g/kg and 10 g/kg of lysed yeast *S. cerevisiae*, (4) ASB: CON + 1.5 g/kg and 1 g/kg of acidifier sodium butyrate.

^c^Pooled standard error of the mean.

### Diarrhea incidence

There was a trend to reduce the diarrhea incidence (*P* = 0.098) in pigs fed NUC diet and improved fecal score (*P* = 0.090) in pigs fed YSC diet than pigs fed CON diet in the phase 1 (Table 2). In phase 2, there was a trend to reduce the diarrhea incidence (*P* = 0.089) and improved fecal score (*P* = 0.091) in pigs fed YSC diet than in those fed CON diet.

**Table 2:** Diarrhea incidence and fecal score of piglets fed diets supplemented or not with feed additives (n = 9 pens replicate per dietary treatment and 5 piglets per pen as an experimental unit).

Item	Dietary treatment^a^				SEM^b^	*P*-value		
	CON	NUC	YSC	ASB		CON × NUC	CON × YSC	CON × ASB
Phase 1								
Diarrhea incidence, %	20.01	12.10	14.33	15.56	3.28	0.098	0.230	0.345
Fecal score	0.80	0.64	0.61	0.69	0.07	0.161	0.090	0.341
Phase 2								
Diarrhea incidence, %	15.50	16.00	6.70	7.16	3.55	0.927	0.089	0.106
Fecal score	0.53	0.60	0.32	0.37	0.11	0.516	0.091	0.193

^a^Dietary treatment: phase 1 (24–32 days) and phase 2 (32–45 days), respectively: (1) CON: control, basal diet, (2) NUC: CON + 1 g/kg and 0.5 g/kg of nucleotides, (3) YSC: CON + 20 g/kg and 10 g/kg of lysed yeast *S. cerevisiae*, (4) ASB: CON + 1.5 g/kg and 1 g/kg of acidifier sodium butyrate.

^b^Pooled standard error of the mean.

### Blood profile

There was no effect of dietary treatments on IgG, creatinine, and urea concentrations (Table 3).

**Table 3:** Blood profile of piglets fed diets supplemented or not with feed additives on d 32 (n = 9 pigs replicates per dietary treatment).

Item	Dietary treatment^a^				SEM^b^	*P*-value		
	CON	NUC	YSC	ASB		CON × NUC	CON × YSC	CON × ASB
Urea, mg/dL	16.55	16.89	15.55	13.11	1.58	0.882	0.658	0.133
Creatinine, mg/dL	0.64	0.70	0.68	0.66	0.06	0.416	0.330	0.458
IgG, mg/dL	272.74	306.67	266.07	273.69	23.42	0.157	0.777	0.968

^a^Dietary treatment: *CON* control, basal diet, *NUC* CON + 1 g/kg of nucleotides, *YSC* CON + 20 g/kg of lysed yeast *S. cerevisia*, *ASB* CON + 1.5 g/kg of acidifier sodium butyrate.

^b^Pooled standard error of the mean.

### Intestinal morphology

Dietary treatments had no effects on villus height, crypt depth, villus:crypt ratio, and proportion of goblet cells in the duodenum and jejunum (Table 4). In the ileum, the dietary treatments had no effects on crypt depth and proportion of goblet cells. However, villus height was increased (*P* < 0.05) and villus:crypt ratio trended to increase (*P* = 0.066) in pigs fed ASB diet than pigs fed CON diet. In addition, the number of Peyer’s patches in pigs fed YSC or ASB diets was lower (*P* < 0.05) compared to pigs that received CON diet. There was a trend towards an increase (*P* = 0.071) villus height and reduce (*P* = 0.078) the number of Peyer’s patches in pigs fed NUC diet than those fed the CON diet.

**Table 4:** Intestinal morphology of piglets fed diets supplemented or not with feed additives on d 32 (n = 9 pigs replicates per dietary treatment).

Item	Dietary treatment^a^				SEM^b^	*P*-value		
	CON	NUC	YSC	ASB		CON × NUC	CON × YSC	CON × ASB
Duodenum								
Villus height, μm	307	297	298	300	19.09	0.662	0.689	0.753
Crypt depth, μm	178	176	182	177	9.23	0.840	0.761	0.926
Villus:crypt ratio	1.72	1.70	1.63	1.71	0.08	0.812	0.387	0.857
Goblet cells, %	32.50	34.25	33.51	36.79	3.00	0.656	0.790	0.227
Jejunum								
Villus height, μm	264	255	261	268	24.09	0.771	0.922	0.880
Crypt depth, μm	150	139	143	157	11.12	0.474	0.640	0.590
Villus:crypt ratio	1.80	1.83	1.82	1.71	0.10	0.804	0.851	0.486
Goblet cells, %	23.15	24.62	25.16	22.79	2.76	0.683	0.562	0.912
Ileum								
Villus height, μm	210	241	234	255	12.99	0.071	0.150	< 0.010
Crypt depth, μm	132	139	138	138	9.17	0.534	0.573	0.576
Villus:crypt ratio	1.64	1.66	1.69	1.81	0.07	0.896	0.603	0.066
Goblet cells, %	50.43	53.07	46.65	45.94	4.59	0.661	0.514	0.405
Peyer’s patches, n	48	40	39	38	3.33	0.078	0.042	0.017

^a^Dietary treatment: *CON* control, basal diet, *NUC* CON + 1 g/kg of nucleotides, *YSC* CON + 20 g/kg of lysed yeast *S. cerevisiae*, *ASB* CON + 1.5 g/kg of acidifier sodium butyrate.

^b^Pooled standard error of the mean.

### mRNA relative abundance of nutrient transporters

**Figure 1:**
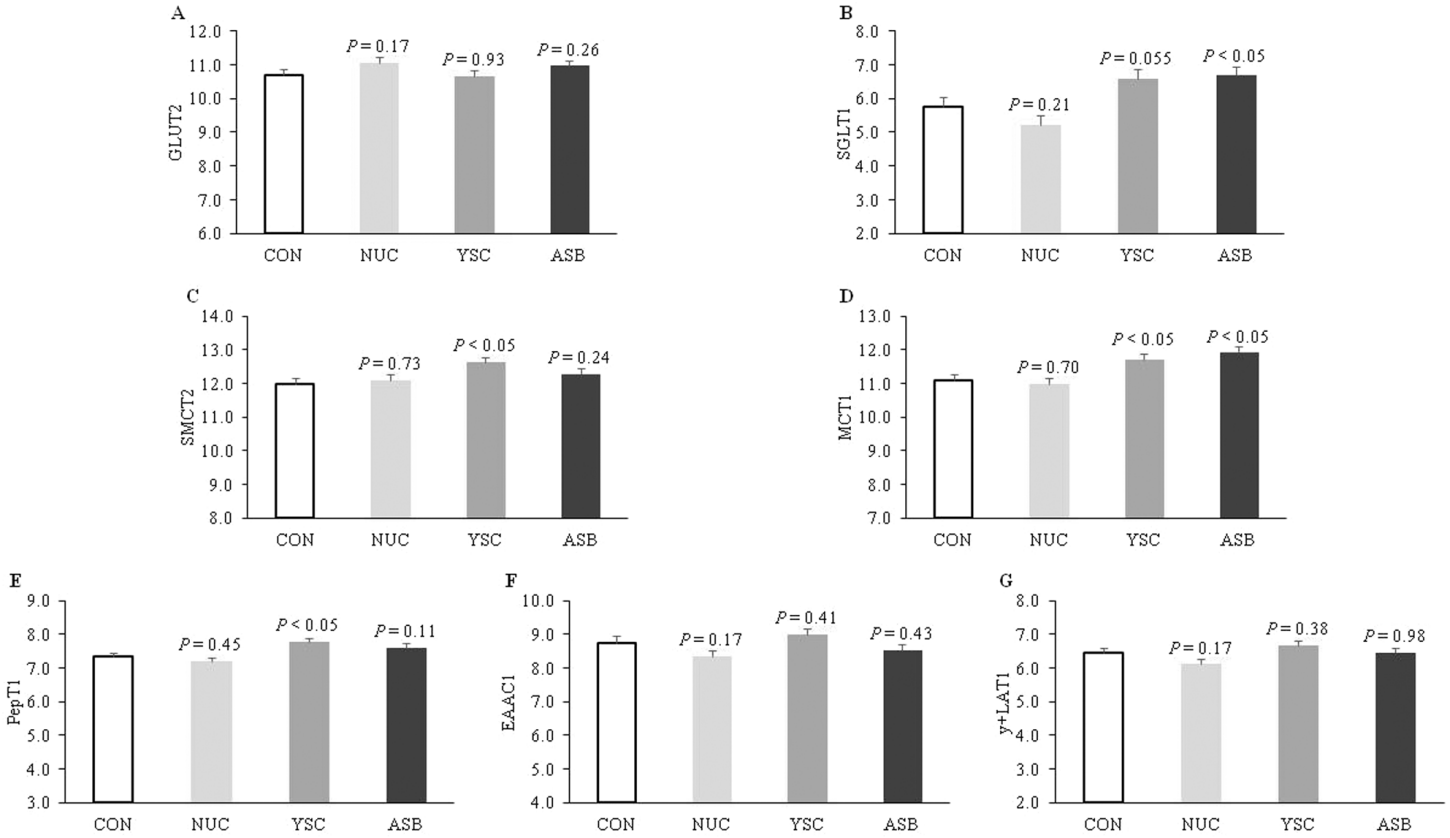
mRNA relative abundance of nutrient transporters in the jejunum of piglets fed diets supplemented or not with feed additives on d 32. Dietary treatment: CON, control, basal diet; NUC, CON + 1 g/kg of nucleotides; YSC, CON + 20 g/kg of lysed yeast *S. cerevisiae*; ASB: CON + 1.5 g/kg of acidifier sodium butyrate. Data are means of 9 pigs replicates per dietary treatment. *P*-value, preplanned contrasts of each of the treatments (NUC, YSC, and ASB) versus the control treatment (CON). (**A**) GLUT2, glucose transporter type 2; (**B**) SGLT1, Na^+^/glucose co-transporter 1; (**C**) SMCT2, sodium-coupled monocarboxylate transporter; (**D**) MCT1, monocarboxylate transporter 1; (**E**) PepT1, peptide transporter 1; (**F**) EAAC1, excitatory amino acid carrier 1; (**G**) y^+^LAT1, Na^+^-independent cationic and Na^+^-dependent neutral amino acid transporter 1.

In the jejunum, there was no effect of dietary treatments on the mRNA expression of GLUT2, EAAC1, and y^+^LAT1 (Fig. 1). However, mRNA expression of SMCT2 and PepT1 was higher (*P* < 0.05) in pigs fed YSC diet compared to pigs fed CON diet. The mRNA expression of MCT1 in pigs fed ASB and YSC diets was higher (*P* < 0.05) than in those fed CON diet. In addition, SGLT1 mRNA expression in pigs fed ASB diet was higher (*P* < 0.05), and YSC diet (*P* = 0.055) trended upwards compared to pigs fed CON diet.

### mRNA relative abundance of inflammatory and antioxidants markers, and tight junction proteins

**Figure 2:**
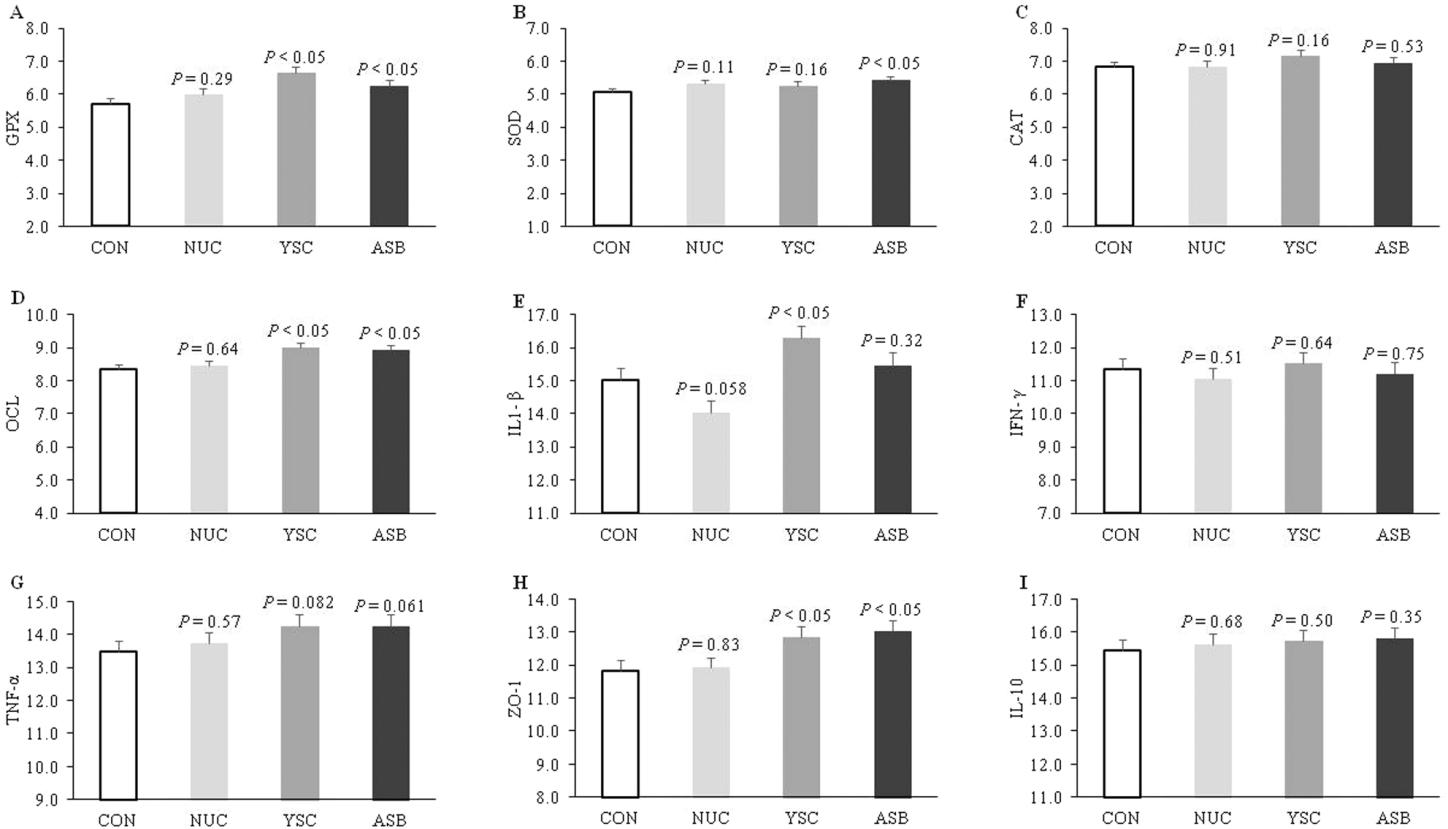
mRNA relative abundance of inflammatory and antioxidants markers, and tight junction proteins in the jejunum of pigls fed diets supplemented or not with feed additives on d 32. Dietary treatment: CON, control, basal diet; NUC, CON + 1 g/kg of nucleotides; YSC, CON + 20 g/kg of lysed yeast *S. cerevisiae*; ASB: CON + 1.5 g/kg of acidifier sodium butyrate. Data are means of 9 pigs replicates per dietary treatment. *P*-value, preplanned contrasts of each of the treatments (NUC, YSC, and ASB) versus the control treatment (CON). (**A**) GPX, glutathione peroxidase; (**B**) SOD, superoxide dismutase; (**C**) CAT, catalase; (**D**) OCL, occludin; (**E**) ZO-1, zonula occludens-1; (**F**) IFN-γ, interferon gamma; (**G**) TNF-α, tumor necrosis factor alpha; (**H**) IL1-β, interleukin 1 beta; (**I**) IL-10, interleukin 10.

There was no effect of dietary treatments on the mRNA expression of CAT, IFN-γ, and IL-10 (Fig. 2). However, mRNA expression of GPX, OCL, and ZO-1 was higher (*P* < 0.05) in pigs fed YSC or ASB diets compared to pigs fed CON diet. The mRNA expression of SOD in pigs fed ASB diet, and IL1-β in pigs fed YSC diet was higher (*P* < 0.05) than in those fed CON diet. In addition, TNF-α mRNA expression in pigs fed YSC diet (*P* = 0.082) and ASB diet (*P* = 0.061) trended upwards compared to pigs fed CON diet. There was a downward trend (*P* = 0.058) in IL1-β mRNA expression of pigs fed NUC diet than pigs that received CON diet.

## Discussion

The hypothesis of the current experiment was that the dietary supplementation of purified nucleotide, *S. cerevisiae* yeast, or encapsulated acidifier sodium butyrate would improve the growth performance of weaned piglets and beneficially affect gut parameters. The present results showed that feeding nursery pigs with YSC or ASB diets increased growth performance and improved gut health, whereas no improvements were observed feeding the NUC diet.

Although nucleotides can be synthesized from other precursors in pigs’ diets, in the post-weaning a stressful and limited nutrient intake period, nucleotides could be considered an essential nutrient^6^. However, in the present study, performance was not influenced by NUC diet, while others have reported positive effects of nucleotide supplementation on piglet growth performance^26,27^ and feed intake^5^. The NUC diets correspond to 100 mg nucleotides/kg diet in phase 1 and 75 mg nucleotides/kg in phase 2 in a 24-day trial and were fed purified. On the other side, Superchi et al.^26^ used a nucleotide yeast-derived source that also contains viable cells, cell wall components, inositol, and functional amino acids. Weaver and Kim^27^ demonstrated improved growth performance with up to 1000 mg nucleotides/kg during a 28-day trial. Working with different levels of nucleotides (0, 50, 150, 250, and 500 mg/kg) in a 20-day trial with one pig per pen, Jang and Kim^5^ only found significant improvements in the feed intake, and when feeding the lower supplementation levels (50 and 150 mg/kg). Thus, the discrepancies in growth performance results between studies are justified due to the different sources, the different dosages used, and administration time.

Regarding the YSC diet, the improved growth performance results are attributed to the improvements in intestinal health promoted by the supplementation of *S. cerevisiae* yeast. This additive contains high amounts of highly digestible protein, essential amino acids, nucleotides, mannanoligosaccharides, and β-glucans^15^. These components present in yeast may have anti-inflammatory properties to reduce intestinal inflammation and, consequently, minimize diarrheal disorders (as observed in the current study) and nutrient malabsorption^1^. According to Kogan and Kocher^19^, β-glucans are capable of blocking fimbriae of pathogenic bacteria and preventing their adhesion to the epithelium of the intestinal mucosa, acting to prevent or eliminate infection. Yeast-based additives support the immune system of piglets by modulating the intestinal microbiota^28^, which may contribute to improve growth performance^29^.

An increase in nutrient digestibility as reported by Barbosa et al.^30^ and Boontiam et al.^8^ is another mechanism by which yeast supplementation in nursery pigs’ diets can improve the growth performance. Nutrient transporters are proteins expressed in the apical membrane of intestinal cells that absorb nutrients and, therefore, it is possible to improve digestibility by increasing the expression of these transporters^31^. This is supported by the results of our study, which showed increased mRNA expression of nutrient transporters (SGLT1, SMCT2, MCT1, and PepT1) in the jejunum of pigs. The SGLT1 is responsible for glucose absorption, while PepT1 acts on peptide absorption. The increase in the expression of these transporters suggested that there was greater availability of glucose and amino acids at the cellular level, in agreement with the findings by Clarke et al.^31^. Similarly, the increased expression of SMCT2 and MCT1 indicated a greater availability of monocarboxylates (e.g. lactate, short-chain fatty acids, and ketone bodies)^32^, which represent substrates for maintaining the energetic state in cells like the enterocytes.

Regarding the ASB diet, the improvement in growth performance is supported by the increase in SGLT1 and MCT1 mRNA expression in the jejunum of nursery pigs. The MCT1 is expressed in both the small and large intestines, and its function is to transport butyrate into the cell^33^. Although, in the small intestine, microbial butyrate formation is low or absent^34^, the addition of butyrate to diets exerts trophic effects^35^ and stimulates the secretion of digestive enzymes^21^, and this can result in more efficient digestion and absorption of dietary nutrients, leading to improved performance. Furthermore, butyrate is a source of energy for enterocytes and additionally has an antiapoptotic effect^33^. Therefore, greater MCT1 and SGLT1 expression may enhance the absorption of nutrients and energy to cope with post-weaning stress^36^. In the present study, sodium butyrate was added in encapsulated form (composed of a vegetable fat-based coating material) to be enzymatically broken down by lipase secreted in the duodenum, as mentioned by Maito et al.^10^. According to Tugnoli et al.^25^, the encapsulation process provides protection that allows a gradual release of the acidifier along the length of the gastrointestinal tract, reducing the dissociation in the stomach and maintaining their efficacy in jejunum and ileum.

The weaning transition promotes physiological changes in the structural and functional aspects of the intestine, causing villous atrophy and increased crypt depth, which in turn reduces the small intestine capacity to absorb nutrients^3^. In the present study, villus height and villus:crypt ratio in the ileum were higher in pigs fed the ASB diet, indicating improvement in intestinal morphology. Moreover, a greater abundance of jejunal mucosal barrier function-related genes (OCL and ZO-1) was observed in pigs fed the ASB diet. These results indicated that ASB diet was efficient in the structural maintenance of the small intestine, as suggested by others^35^. The gradual release of sodium butyrate throughout the intestinal tract seems to be critical in achieving the expected target^9^, allowing the additive to affect different portions of the intestine such as jejunum and ileum. According to Guilloteau et al.^33^, supplementation with sodium butyrate stimulates the proliferation of epithelial cells, resulting in a greater absorption surface and preservation of villi length, reflecting the improvements observed in growth performance of pigs.

A greater abundance of jejunal OCL and ZO-1 mRNA expression was also observed in pigs fed the YSC. According to Rose et al.^37^, OCL and ZO-1 are classified as intestinal junction proteins with a role in regulating epithelial permeability. Decreased intestinal permeability can be correlated with reduced oxidative stress, because this results in attenuation of damage to the intestinal mucosal barrier^14^. Oxidative stress is a physiological stage in which antioxidant defense is inadequate to detoxify the reactive oxygen species, this oxidative process damages essential biomolecules, leading to reduced growth performance^29^. The GPX and SOD as the key enzymes of the antioxidant system play a crucial role in eliminating free radicals, reducing oxidative damage, and maintaining cell structure is well known^38^. In our study, in addition to the increase in the expression of tight junction proteins, pigs fed the ASB and YSC diet had greater expression of GPX and SOD mRNA. These results suggested that nursery pigs fed with additives had greater antioxidant capacity than those fed the CON diet, demonstrating that there was an improvement in the intestinal redox state.

The impacts of weaning stress are not only limited to intestinal barrier function and oxidative stress, but an increase in the activation of the immune system in weaned piglets is also observed^3^. An unregulated enhanced immune response may trigger a negative effect on other metabolic processes and as a result impair growth performance^39^. Peyer's patches are the major organized lymphoid structures involved in the induction of mucosal immune responses in the intestine^40^. In the present study, a reduction in Peyer's patch counts was observed in the ileum of pigs fed YSC or ASB diet. This suggested that there was less induction of the mucosal immune system in pigs that received YSC and ASB diets and, consequently, less stimulus to the immune system because Peyer's patches can be considered as immunological sensors of the intestine^41^. These results suggested that both yeast and sodium butyrate could help enhance the small intestine epithelial barrier, antioxidant capacity, and immune system. Thus, the enhancement of overall gut health helps explain the improvement in the growth performance of pigs fed YSC and ASB diets.

Regarding the production of pro-inflammatory cytokines, it was found that pigs fed the YSC diet showed an increase in IL1-β mRNA expression and a tendency to increase TNF-α, while piglets fed the ASB diet showed a tendency to increase TNF-α mRNA expression. On the other hand, pigs fed the NUC diet had a tendency to reduce IL1-β mRNA expression. Nucleotides, yeast, and acidifier-based additives can improve pig immune responses in the post-weaning period^16^. These additives can activate immune cells, including macrophages, which produce IL-1β as part of the immune response^42,43^. The results of the present study indicated a sustained condition of immune response, because in some cases, a controlled and transient increase in IL-1β may be part of a healthy immune response to support the ability of pigs to fight infections or maintain intestinal health^18^. According to Grimble^39^, it is considered beneficial the presence of cytokines (e.g. IL-1β and TNF-α) in adequate concentrations during an inflammatory response to infection. It is important to avoid overstimulation of the immune system, as greater expression of pro-inflammatory cytokines can trigger pathological responses in inflammatory conditions^44^. However, in a previous study, it was observed that TNF-α increases the expression of specific anti-apoptotic proteins, as well as triggers the expression of the survival gene BCL2A1 (not evaluated in the current study) in the intestine of weaned piglets^45^. Also, the β-glucans present in yeast are recognized by specific receptors (pattern recognition receptors) on immune cells, such as macrophages and neutrophils. In particular, they are recognized by the dectin-1 receptor^46^. Once β-glucans bind to these receptors, they can trigger an immune response. Upon recognition of β-glucans, immune cells can produce pro-inflammatory cytokines, such as TNF-α, and IL-1β^47^. Collectively, although these cytokines play a central role in initiating and magnifying the inflammatory response, they did not negatively affect the biological response of nursery pigs fed YSC and ASB diets.

## Conclusions

Based on the evaluated criteria, dietary supplementation of autolyzed yeast *S. cerevisiae* or sodium butyrate promotes better growth performance by improving the integrity of the intestinal barrier, the mRNA expression of nutrient transporters and antioxidant enzymes in the jejunum of nursery pigs, but without major differences in intestinal morphology in those fed with *S. cerevisiae* yeast. Furthermore, none of the dietary treatments promoted changes in the observed blood metabolites, but a diet containing *S. cerevisiae* yeast or sodium butyrate provided a sustained immune response in the jejunum of nursery pigs. On the other hand, dietary nucleotide supplementation did not improve growth performance and gut health.

## Material and methods

The experimental protocol follows the ethical principles in animal research (CONCEA, 2016) and was approved by the Ethical Committee on Animal Use of Universidade Federal de Viçosa, under protocol no. 0110/2022. All methods were carried out in accordance with relevant guidelines and regulations. All methods were reported in accordance with ARRIVE guidelines (https://arriveguidelines.org/arrive-guidelines).

### Animals, experimental design, housing, and diets

The experiment was conducted on a commercial farm in the municipality of Santo Antônio do Grama, MG, Brazil. A total of 180 piglets [PIC 337 (Large White × Landrace × Duroc × Pietrain) × DB 90 (Large White × Landrace)] castrated males and females, weaned at 21 day-old and with 5.17 ± 0.57 kg BW were used in a 24-day trial. Piglets were assigned to a randomized block design based on their initial BW (light 4.7 ± 0.25 kg and heavy 5.6 ± 0.34 kg) into 4 dietary treatments and 9 pens replicates (5 pigs/pen).

The experiment was conducted only in one series, without an adaptation period. The pigs were housed in suspended pens (1.75 × 1.00 m, 0.35 m^2^/pig), with a plastic floor, semi-automatic feeders and nipple drinkers, with free access to diet and water. The minimum and maximum temperatures in the nursery room were 22.9 ± 1.50 °C and 31.8 ± 2.54 °C, respectively.

Pigs were fed in a two-phase feeding regimen (phase 1: 21–32 days, and phase 2: 32–45 days). All diets were corn and soybean meal-based with industrial amino acids and formulated according to the nutritional recommendations of the Brazilian Tables for Poultry and Swine^48^ (Table 5), and provided in mash form. During phases 1 and 2, the dietary treatments consisted, respectively, of: (1) CON: control, basal diet, (2) NUC: CON + 1 g/kg and 0.5 g/kg of nucleotides, (3) YSC: CON + 20 g/kg and 10 g/kg of lysed yeast *S. cerevisiae*, (4) ASB: CON + 1.5 g/kg and 1 g/kg of acidifier sodium butyrate. Feed additives were added in replacement with inert in the CON based on the manufacturer's recommendations.

**Table 5:** Ingredients and chemical composition of control diets fed to nursery piglets (g/kg, as-fed basis).

Item^a^	Phase 1	Phase 2
	21–32 days	32–45 days
Ground corn, 7.8% CP	388.1	470.9
Soybean meal, 46.0% CP	219.7	242.2
Dried whey, 12.5% CP	150.0	100.0
Soybean micronized, 36.0% CP	100.0	70.0
Extrude corn, 7.6% CP	20.0	20.0
Plasma protein, 78.0% CP	20.0	10.0
Sugar	30.0	30.0
Inert (kaolin)	20.0	10.0
Dicalcium phosphate	9.35	10.6
Limestone calcitic	7.69	7.34
Soybean oil	15.93	11.1
Zinc oxide	2.50	2.20
Choline chloride	1.95	1.50
L-lys, 78.0%	4.69	4,45
DL-met, 99.0%	2.32	1.96
L-thr, 98.5%	2.30	2.14
L-trp, 99.0%	0.32	0.28
L-val, 96.5%	1.03	0.76
Salt	1.90	2.44
Copper sulfate	0.60	0.60
Vitamin-mineral premix^b^	1.40	1.40
Phytase^c^	0.05	0.05
BHT	0.10	0.10
Calculated composition		
ME, kcal/kg	3400	3375
Crude protein, g/kg	213.00	213.00
SID^d^ lys, g/kg	14.50	13.46
SID met, g/kg	4.90	4.53
SID met + cys, g/kg	8.13	7.54
SID thr, g/kg	9.72	9.02
SID trp, g/kg	2.76	2.56
SID val, g/kg	10.01	9.29
SID ile, g/kg	8.01	7.70
Total Ca, g/kg	8.50	8.25
Available P, g/kg	5.19	5.00
Total Na, g/kg	2.80	2.30
Lactose, g/kg	112.50	75.00

^a^Feed additives were included as a replacement for the inert in the diet. Phase 1 (21 to 32 d) and phase 2(32 to 45 d), respectively: (1) CON: control, basal diet, (2) NUC: CON + 1 g/kg and 0.5 g/kg of nucleotides (Nucleobase 1.5, Aleris Animal Nutrition, Jundiaí, SP, Brazil), (3) YSC: CON + 20 g/kg and 10 g/kg of lysed yeast *S. cerevisiae* (Sinergis, Aleris Animal Nutrition, Brazil), (4) ASB: CON + 1.5 g/kg and 1 g/kg of acidifier sodium butyrate (Nuttritiva B90CNa, Additiva Nutrition, Monte Alegre do Sul, SP, Brazil).

^b^Composition per kg of diet: vitamin A, 12,000 IU; vitamin D_3_, 2,250 IU; vitamin E, 65 IU; vitamin K, 3 mg; thiamine, 2.25 mg; riboflavin, 6 mg; pyridoxine, 2.25 mg; vitamin B_12_, 27 mcg; folic acid, 400 mcg; biotin, 150 mcg; pantothenic acid, 22.5 mg; niacin, 45 mg; copper sulfate, 10 mg; iodine, 1.5 mg; iron sulfate, 100 mg; manganese sulfate, 40 mg; sodium selenite, 0.3 mg; zinc oxide, 100 mg.

^c^Natuphos®, Basf enzyme.

^d^Standardized ileal digestible.

### Composition of feed additives

The source of nucleotides (Nucleobase 1.5, Aleris Animal Nutrition, Jundiaí, SP, Brazil) obtained from yeast extract purified with autolyzed yeast, contained 15% free nucleotides and 85% vehicle, corresponding to 100 mg free nucleotides/kg diet (phase 1) and 75 mg free nucleotides/kg (phase 2). The yeast source (Sinergis, Aleris Animal Nutrition, Brazil) contained 100% autolyzed yeast *S. cerevisiae.* The acidifier source (Nuttritiva B90CNa, Additiva Nutrition, Monte Alegre do Sul, SP, Brazil) contained 90% sodium butyrate in the form of a protected and encapsulated salt with palm oil, microgranulated, corresponding to 1.35 g sodium butyrate/kg diet (phase 1) and 0.90 g sodium butyrate/kg diet (phase 2).

### Growth performance and diarrhea incidence

Throughout the trial, the offered diet and leftovers were weighed to calculate average daily feed intake (ADFI). Pigs were individually weighed on d 21, 32, and 45 to determine BW, average daily weight gain (ADG), and feed conversion (FC). The fecal consistency of each pig was visually assessed during phase 1 (24–32 days) and phase 2 (32–45 days), using the method described by Liu et al.^49^. Fresh feces were ranked on a 4-point scale as follows: 0 = solid, 1 = semi-solid, 2 = semi-liquid, and 3 = liquid. The diarrhea incidence was defined as the consistency of feces at scale 2 or 3 for 2 continuous days. The diarrhea incidence for the pigs was calculated as follows: diarrhea incidence (%) = [(the number of pigs with diarrhea in each pen × number of days of diarrhea) ÷ (total number of pigs in each pen × number of days)] × 100%. Observations were made in the morning, every day throughout the experimental period by a trained evaluator.

### Sample collection

At 32 day-old, blood was collected from 1 piglet with BW closest to the average weight of the pigs within its respective pen. The animals were not fasted. Blood was collected (09h00) by orbital sinus puncture with a hypodermic needle (40 × 1.6 mm) into 10 mL tubes without anticoagulants. Samples were immediately sent at room temperature to the Viçosa Clinical Laboratory (Viçosa, MG—Brazil) for determination of serum urea nitrogen (SUN; Ureal Cobas C311, Linklab, software PNCQ), creatinine (Creatinine, WS-Kovalent, kinetic method, ASB-380, Mindray), and immunoglobulin G concentrations (IgG Atellica CH IgG_2, CH Analyzer, Siemens Healthineers).

The same blood donor piglet was electrically stunned (240 V for 3 s) followed by exsanguination to collect samples on d 32. Fragments measuring 2 cm were sampled (8 pigs/treatment) from the duodenum (10 cm from the pylorus junction), jejunum (mid-section), and ileum (5 cm to ileocecal junction) for histological evaluation^50^. The histological sections were then washed in a physiological solution (0.9% sodium chloride) and fixed in 4% paraformaldehyde solution (100 mL 40% paraformaldehyde, 900 mL distilled water, 2.28 g monobasic sodium phosphate, and 21.74 g dibasic sodium phosphate) for 24 h at room temperature^6^. Another 2 cm of jejunum was collected and immediately frozen in liquid nitrogen, stored at − 80 °C for RNA extraction and gene expression analysis.

### Intestinal morphology, Peyer’s patches, and goblet cells

After 24 h of fixation, the fragments of the duodenum, jejunum, and ileum were transferred to an ethanol solution 70% (v/v). Then, the samples were cut into cross-sections and dried in increasing gradients of ethyl, diaphanized in HistoChoice®, and embedded in liquid Paraplast® at 65 °C. Five cross-sections (5 μm thickness each) were placed per slide and stained with hematoxylin and eosin. The sections were semi-serial using 1 in 10 cuts^51^. For morphological readings of villus height and crypt depth in the duodenum, jejunum, and ileum, an EVOS™ M5000 Cell Imaging System optical microscope (Invitrogen, Thermo Fisher Scientific) with a 10-objective lens was used. The images were then analyzed using ImageJ 1.50i (Java1.6.0_20; National Institutes of Health, USA). Heights of 20 villus and their 20 crypts were selected and measured. Villus to crypt ratios using the length data were then calculated. All measurements were made by a single trained individual. In the ileum fragment, the total count of the Peyer’s patches was performed at 4 × magnification^52^.

To assess the goblet cells in the duodenum, jejunum, and ileum, 10 fields per slide were photographed at 20 × magnification. Subsequently, the ImageJ program was used, and perpendicular lines were inserted with markings in uniformly sized quadrants under each image. Then, the total number of intersections in the image and the cells that touched the intersections were counted. The calculation was made according to the methodology proposed by Mandarim de Lacerda^53^:

$$\text{Goblet cells }\left(\text{\%}\right)=\frac{total\,number\,of\,goblet\,cells \times 100}{total\,number\,total\,number\,of\,intersections}$$

### Relative mRNA abundance

Total RNA was extracted using a commercial kit (SV Total RNA isolation kit—Promega, Z3100), following the manufacturer's instructions. The RNA concentration was estimated using NanoDrop™ Lite (Thermo Fisher Scientific), and RNA integrity was assessed using 1% agarose gel electrophoresis. Complementary DNA was synthesized according to the GoScript™ Reverse Transcription System protocol (Promega Corporation). GenBank numbers used to access the gene primers are shown in Table 6. Primers were used for reverse transcription quantitative PCR with GoTaq® qPCR Master Mix (Promega) in QuantStudio® 3 (Applied Biosystems, Thermo Fisher Scientific). Geometric mean of Ct value of β-actina was used to normalize the expression of the target genes for the jejunum samples. The relative expression of the gene of interest was calculated by ΔCt^54^ for glutathione peroxidase (GPX), superoxide dismutase (SOD), catalase (CAT), occludin (OCL), zonula occludens-1 (ZO-1), interferon gamma (IFN-γ), tumor necrosis factor alpha (TNF-α), interleukin 1 beta (IL1-β), interleukin 10 (IL-10), glucose transporter type 2 (GLUT2), Na^+^/glucose co-transporter 1 (SGLT1), sodium-coupled monocarboxylate transporter (SMCT2), monocarboxylate transporter 1 (MCT1), peptide transporter 1 (PepT1), excitatory amino acid carrier 1 (EAAC1), Na^+^-independent cationic and Na^+^-dependent neutral amino acid transporter 1 (y^+^LAT1).

**Table 6:** List of primers used in reverse transcription quantitative-PCR gene expression analysis in weaned piglets.

Genes	GenBank number	Sequence^2^
GPX	NM_214201.1	F: 5′GCCCAACTTCATGCTCTTC3′
		R: 5′CAGGATCTCCCCATTCTTGGC3′
SOD	NM_001190422.1	F: 5′ATCAAGAGAGGCACGTTGGA3′
		R: 5′TCTGCCCAAGTCATCTGGTT3′
CAT	NM_214301.2	F: 5′GCTTTAGTGCTCCCGAACAG3′
		R: 5′AGATGACCCGCAATGTTCTC3′
OCL	NM_001163647.1	F: 5′TCCTGGGTGTGATGGTGTTC3′
		R: 5′CGTAGAGTCCAGTCACCGCA3′
ZO-1	XM_003353439.2	F: 5´AAGCCCTAAGTTCAATCACAATCT3′
		R: 5´ATCAAACTCAGGAGGCGGC3′
IFN- γ	NM_213948	F: 5′TGGTAGCTCTGGGAAACTGAATG3′
		R: 5′GGCTTTGCGCTGGATCTG3′
TNF-α	NM_214022.1	F: 5′CATCGCCGTCTCCTACCA3′
		R: 5′CCCAGATTCAGCAAAGTCCA3′
IL1- β	NM_214055.1	F: 5′TCTGCCCTGTACCCCAACTG3′
		R: 5′CCCAGGAAGACGGGCTTT3′
IL-10	NM_214041.1	F: 5′GAAGGACCAGATGGGCGACTT3′
		R: 5′CACCTCCTCCACGGCCCTTG3′
GLUT2	FJ209732.1	F: 5′TTGCCTTGGATGAGTTATGTGA3′
		R: 5′GCGTGGTCCTTGACTGAAAA3′
SGLT1	MW_280290.1	F: 5′GGTTGGAGCTTCTCTGTTTG3′
		R: 5′CCAGAACAACCACCCAAATC3′
SMCT2	XM_003122908.1	F: 5′AGGTCTACCGCTTTGGAGCAT3′
		R:5′GAGCTCTGATGTGAAGATGATGACA3′
MCT1	AM_286425.1	F: 5′GGTGGAGGTCCTATCAGCAG3′
		R: 5′AAGCAGCCGCCAATAATCAT3′
PepT1	AY_180903.1	F: 5′CATGGATGCTGTGGTGTATC3′
		R: 5′CATGGAAGCCAGGAACATC3′
EAAC1	NM_001164649.1	F: 5′CAGTGGATGCCATGTTAGAC3′
		R: 5′CGTCTCTGGCTCACTAGAA3′
y^+^LAT1	EU_047705.1	F: 5′TGCGTGGAGGACATCTT3′
		R: 5′CTCCTTCCAGCGCAAATAG3′
β-actin	U07786.1	F: 5′CTCTTCCATCGTGTCCTTCTAC3′
		R: 5′CCTCAGACTTGTCGATCTTCTG3′

*GPX* glutathione peroxidase, *SOD* superoxide dismutase, *CAT* catalase, *OCL* occluding, *ZO-1* zonula occludens-1, *IFN-γ* interferon gamma, *TNF-α* tumor necrosis factor alpha, *IL1-β* interleukin 1 beta, *IL-10* interleukin 10, *GLUT2* glucose transporter type 2, *SGLT1* Na + /glucose co-transporter 1, *SMCT2* sodium-coupled monocarboxylate transporter, *MCT1* monocarboxylate transporter 1, *PepT1* peptide transporter 1, *EAAC1* excitatory amino acid carrier 1, *y* + *LAT1* Na + -independent cationic and Na + -dependent neutral amino acid transporter 1. 2F and R indicate Forward and Reverse primers, respectively.

### Statistical procedures

The pen was considered the experimental unit for growth performance and diarrhea incidence analysis. One pig from each pen was considered the experimental unit for intestinal morphology, gene expression, and blood profile. The statistical model included the fixed effect of dietary treatment, and block and residual error as random factors. The normality of experimental errors was evaluated using Shapiro–Wilk. The data were analyzed using the mixed procedure of SAS 9.4 (SAS Inst., Inc., Cary, NC, USA) via one-way analysis of variance (ANOVA). When an effect was detected in the ANOVA (*P* < 0.05), differences between means were determined by the preplanned contrasts, in which each of the treatments (NUC, YSC, and ASB) were compared versus the control treatment (CON). The statistical significance and tendency were declared at *P* < 0.05 and 0.05 ≤ *P* < 0.10, respectively.

## Data Availability

The datasets used and/or analysed during the current study available from the corresponding author on reasonable request.
